# Augmented reality navigation for precise implantation of LC2 pelvic tunnel screws in minimally invasive surgery

**DOI:** 10.1016/j.fmre.2024.05.010

**Published:** 2024-05-28

**Authors:** Jie Zang, Zhipeng Lin, Chaowei Ding, DongXiao Bian, Junjun Pan, Xiaodong Tang

**Affiliations:** a Musculoskeletal Tumor Department, Peking University People's Hospital, Beijing 100044, China; b State Key Laboratory of Virtual Reality Technology and Systems, Beihang University, Beijing 100191, China

**Keywords:** Augmented reality, Surgical navigation, 3D corner detection, LC2 pelvic tunnel screws, Minimally invasive surgery

## Abstract

**Background:**

Due to the considerable risk of trauma, traditional open surgery has been gradually replaced by minimally invasive surgery using tunnel screws. However, during minimally invasive pelvic surgery, surgical navigation can be improved by effectively using the augmented reality technique. This can further improve the accuracy of the surgery and reduce risks.

**Methods:**

This study adopts a registration approach based on the patient coordinate system and uses a specific manual setting comparison method to select the suitable 3D corner detection parameters.

**Results:**

After experiments, this strategy can effectively improve the accuracy of positioning during surgery. It achieves a balance in detection time and accuracy, which further reduces risks and injuries during surgery.

**Conclusions:**

Our technique can achieve more precise and safer results during minimally invasive pelvic surgery, which shows its great value and potential in the clinic.

## Graphical abstract

**Figure:**
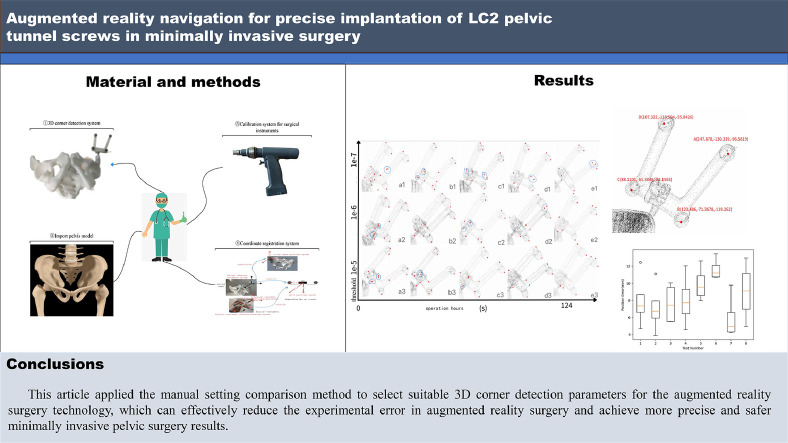

## 1 Introduction

Traditional open surgery is a highly invasive and dangerous operation. In recent years, with the rise in minimally invasive surgery, it has been gradually replaced by minimally invasive surgery whenever possible. Compared to traditional open surgery, minimally invasive pelvic surgery has the advantages of less trauma, less bleeding, and a faster postoperative recovery, and it has been widely used in the treatment of pelvic fractures or tumors [1].

**Fig. 1:**
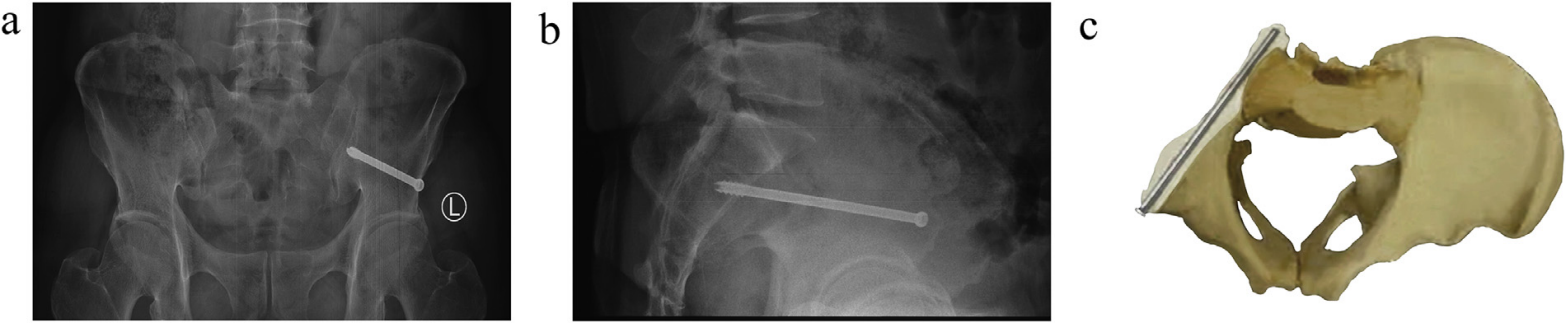
**(a) Pelvis X-ray (anterior view). (b) Pelvis X-ray (lateral view). (c) Cross-section view of the model**.

There are many bone fixation channels in the pelvis, and inserting screws percutaneously into these channels can stabilize fracture fragments and treat fractures. Among the various injuries that can be caused in the pelvis, lateral compression injury accounts for 80% of pelvic injuries [2]. According to the Young–Burgess classification, LC fractures (lateral compression injury) are divided into three types: LC1, LC2, and LC3. LC2 channel screws can be used for the minimally invasive treatment of LC2 fractures [3]. The LC2 screw passes through the anterior inferior iliac spine and points toward the posterior spine. The screw is inserted into a relatively dense bony channel, which is located at the top of the acetabulum, within the ischial branch, and the ilium on the lateral side of the sacroiliac joint, as shown in Fig. 1. Inserting the screw in this direction can effectively treat LC2 fractures [4].

At present, minimally invasive surgery is performed using traditional X-rays or navigation robotics. During the process of minimally invasive surgery, surgeons cannot directly see the bone structure of the pelvis and the important anatomical structures surrounding the tunnel. When performing surgery using traditional X-ray fluoroscopy, multiple perspectives are required to display the bone tunnel for LC2 screws and determine the entry point and placement angle of the screws. At the same time, the screw placement process requires repeatedly changing the X-ray perspective. The depth and angle of the screw insertion can lead to problems such as prolonged surgery time, increased surgical difficulty, and excessive exposure for doctors and patients to radiation [5].

Although the use of navigation and robots for surgery can improve surgical efficiency, the equipment is expensive, and the operation is complex. The learning period for doctors is also long. As invasive positioning is still required, how to insert screws accurately, quickly, and safely, while reducing medical costs and operation times, is an important issue that still has to be addressed in minimally invasive pelvic surgery [6].

In recent years, augmented reality technology has been increasingly applied in surgery to provide real-time visual imaging information for doctors [7], which not only improves surgical safety, but also considerably improves surgical efficiency and quality. Due to the complex structure of the pelvis, surgeons need to understand three-dimensional anatomical information to accurately and safely complete the surgery [8]. In minimally invasive surgery for pelvic fractures, augmented-reality-assisted percutaneous sacroiliac screws have received considerable attention. It has been confirmed that the use of an augmented-reality-based surgical navigation system can aid in the accurate and safe placement of percutaneous sacroiliac screws [9]. However, there are currently few studies and reports on augmented reality navigation for pelvic LC2 channel screws, resulting in great limitations in its practical application.

This article explores the use of manual setting comparison methods to improve the accuracy of augmented navigation technology during pelvic surgery and ensure both the accuracy of the fluoroscopy box for the minimally invasive placement of LC2 pelvic passage screws and the safety of the surgery. With the improvement of its accuracy, the surgical efficiency is also improved, medical costs are reduced, and the popularity of minimally invasive surgery increases.

## 2 Material and methods

### 2.1 Calibration of the surgical instruments

The key to minimally invasive surgical navigation with pelvic tunnel screws is the real-time tracking of intraoperative surgical instruments. Since optical positioning and tracking instruments can only recognize specific reflective calibration balls, they cannot directly obtain the spatial position and posture of the surgical instruments. In order to track the instruments in real time during the surgery, it is necessary to spatially calibrate the tips of the surgical instruments before the operation to determine the relative spatial relationship between the surgical instruments and fiducial.

The calibration method of the surgical instrument's needle tip requires installing a reflectively marked sphere as a reference coordinate system at the other end of the tip (i.e., the end of the surgical instrument). Therefore, the surgical instruments commonly used in minimally invasive surgery with pelvic channel screws are rigid structures, and the calibration ball holder fixed at the end is also a rigid object. The rigid relationship between the surgical instrument and the calibration ball holder can be solved to obtain the relative position of the tip of the surgical instrument.

### 2.2 Coordinate transformation between the surgical instruments and fiducial

In surgical navigation systems, the essence of the real-time tracking of the surgical instruments is to track the tip of the surgical instrument. In minimally invasive pelvic tunnel screw surgery, the surgical instruments are usually slender, and their tips may bear some resistance when placed in the lesion [10]. Therefore, it is difficult to directly install positioning and tracking sensors of corresponding sizes on the tips of these surgical instruments. In order to address the above problems, in this project, a reflective calibration ball was installed at the end of the surgical instrument as a reference coordinate system. The passive optical positioning method was used to calibrate the needle tip of the surgical instrument to obtain the transformation of the characteristic point of the tip relative to the reference coordinate system. During the operation, the reflective calibration ball can be tracked by the optical positioning and tracking instrument, thereby tracking the position and posture of the tip of the surgical instrument in real time.

**Fig. 2:**
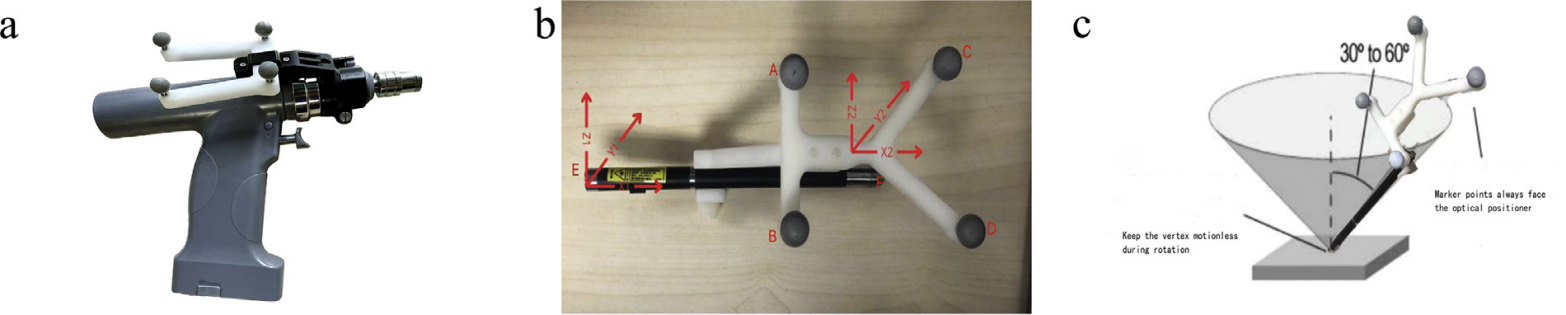
**Surgical instrument and registration system**. (a) Orthopedic bone drill (from Yue da company, Hunan Province, China) with an optical tracking ball. (b) Surgical instrument registration coordinate system. (c) Surgical instrument registration.

The most commonly used surgical instrument to place screws in orthopedic surgery is a bone drill, as shown in Fig. 2a. During the operation, the doctor holds the handle and implants the screws based on the direction of the cylinder above the bone drill. In actual use, the calibration ball should be placed above the bone drill to determine the positional relationship between the calibration ball and the bone drill's needle tip [11]. In this research, the surgical instrument was replaced by a laser pointer to represent the cylindrical part above the bone drill [12]. The laser exit end was set as the tip of the laser pointer. The reflective calibration ball holder was placed at the end of the laser pointer, with four calibration balls according to the fixed structural position composition. Suppose the origins $O_{1}$ and $O_{2}$ are at the end of the laser pointer and the center of fiducial, respectively, and establish a coordinate system centered on $O_{1}X_{1}Y_{1}Z_{1}$ and $O_{2}X_{2}Y_{2}Z_{2}$. This is shown in Fig. 2b.

$O_{1}X_{1}Y_{1}Z_{1}$ represents the coordinate system of the laser pointer tip, and $O_{2}X_{2}Y_{2}Z_{2}$ represents the coordinate system of the calibration ball. The purpose of surgical instrument registration is to determine the deviation relationship between the above two coordinate systems. Optical positioning and tracking instruments are used to track the reflective calibration balls. During calibration, the positions of the laser pointer and the fiducial have a rigid relationship and are fixed. The four calibration balls are represented byA, B, C, and D, and the tip and tail end of the laser pointer are represented by E and F, respectively. $O_{2}$ is at the intersection of AC and BD. The tip coordinate system has origin $O_{2}$; the straight line that passes $O_{2}$ through the same direction as the BA line segment is the Y-axis; the straight line that passes $O_{2}$ perpendicularly to the BA line segment is the X-axis; and the straight line that passes $O_{2}$ perpendicularly to the ABCD plane is the Z-axis. There is only a translational relationship between the two coordinate systems, and there is no rotational offset. Assuming that, in the fiducial coordinate system $O_{2}X_{2}Y_{2}Z_{2}$, the position coordinates of the laser pointer tip are expressed as $X={\left(x,y,z\right)}^{T}$, and the position coordinates in the world coordinates system are expressed as $X_{w}={\left(x_{w},y_{w},z_{w}\right)}^{T}$, the two coordinates have the following correspondence relationship:

(1) $$X_{w}=RX+T$$

It means where R and T, respectively, represent the rotation matrix and translation matrix of the coordinate system in the world coordinate system [13].

### 2.3 Calibration of the surgical instruments

It can be observed from Formula 2.1 that the coordinates in the $O_{2}X_{2}Y_{2}Z_{2}$ fiducial coordinate system need to be solved, which requires a rotation R matrix and a translation T matrix. If the world coordinate system coordinates of the laser pointer tip are unchanged and the laser pointer is rotated to multiple positions, multiple rotation matrices and translation matrices can be obtained. Assuming that the positions are rotated n times during the calibration, and the rotation matrix and translation matrix of each position are recorded as $\left[R_{i},\;T_{i}\right]\left(i=1,2,3,\ldots ,n\right)$, respectively, we obtain the following conversion relationship:

(2) $$X_{w}=R_{1}X+T_{1}=R_{2}X+T_{2}=\ldots =R_{n}X+T_{n}$$

For every rotated position, a correct solution can be obtained. n-1, the average value of the laser pointer offsets, was calculated using the following formula:

(3) $$X={\left(R_{1}-R_{2}\right)}^{-1}\left(T_{2}-T_{1}\right)=\ldots ={\left(R_{n-1}-R_{n}\right)}^{-1}\left(T_{n}-T_{n-1}\right)$$

The average value X is used as the value of the final offset, that is, the O_1_ position in the $O_{2}X_{2}Y_{2}Z_{2}$ coordinate system of the laser pointer tip under the calibration ball. We adopted the root-mean-square deviation (RMSD) of the offset as 0.3 mm, which represents the calibration error, and was calculated using the following formula:

(4) $$RMSD=\sqrt{\frac{\sum_{1}^{n}{\left|X-X_{i}\right|}^{2}}{n}}$$

The accuracy requirements of general surgical navigation systems require that the RMSD value be within 0.3 mm; therefore, it was used as the main basis to assess whether the calibration results could meet the requirements [14].

Combining Eqs. 2 and 3, the following method was used for the calibration: In order to maintain the world $X_{w}$ coordinates unchanged. We placed the tip of the laser pointer at a fixed position and started rotating, so that the optical positioning tracker collects about 300–500 different positions. The rotation matrix and translation matrix are shown in Fig. 2c. In order to always have a field of view of the reflective calibration ball in the AimPosition optical positioning tracker, the rotation angle should be neither too large nor too small, preferably at 30–45° [15]. At the same time, the calibration ball should always face the lens of the optical positioning tracker. For different rotating positions, the AimPosition optical positioning tracker can identify and calculate the rotation matrix $R_{n}$ and translation matrix $T_{n}$ of the reflective calibration ball in the world coordinate system. It uses Eq. 3 to calculate the final offset.

### 2.4 Three-dimensional corner point detection

In order to locate the position of the pelvic model and align the subsequent virtual space with the real space, a fiducial with four calibration balls was placed on the upper right side of the pelvic model. The optical positioning tracker can identify the calibration balls and obtain their spatial coordinate information. The pelvic model and the fiducial are a whole, and a CT scan can be used to scan the whole to reconstruct a 3D model. The 3D Harris algorithm recognition method was used to register the model in the virtual space in line with the real space. Since 3D Harris corners are points with irregularities or sharp changes in value, according to the boundary points and intersections on the surface of an object, and the calibration sphere is a standard sphere with the general characteristics of spherical objects. The calculation process is shown in Fig. S1.

Firstly, to compute the gradient of each point in the three-dimensional point cloud data, that is the normal vector, it is necessary to establish a local coordinate system with the point as the origin and set the size of the spherical space area to fit the plane. The second step uses the obtained gradient value to solve the covariance matrix M. The third step is $R=detM-k{\left(traceM\right)}^{2}$, which is applied to solve the response function R value of this point. The fourth step is to set the T threshold size and use the non-maximum suppression method to filter out the T points corresponding to the larger ones and consider them to be corner points R. Therefore, the normal calculation result contains the feature information of all point clouds contained within the neighboring sphere. The search radius size of 3D Harris corner detection should be set to be the same as the radius of the neighboring sphere. If the radius is too large, the normal information may not cover the internal points in the search area. There may be errors in the cloud data. If the radius is too small, the normal information may include point clouds outside the search area, making the local information inaccurate. The algorithm requires the manual setting of two parameters: the radius K of the fitted spherical spatial region and the threshold T for non-maximum suppression. In subsequent experiments, the experimental results of these parameters were analyzed according the new method.

### 2.5 Spatial registration

In pelvic channel screw surgical navigation, the doctor needs to observe the relative spatial position of the surgical instruments and the pelvic model in real time through the computer screen, as well as the positions of the pelvic structures that the surgical instruments may touch along the current path direction [16]. The key to solve this problem lies in whether the above-mentioned physical spatial relationships can be accurately visualized on the screen. This is the key for doctors to perform surgeries and avoid damaging important anatomical structures [17]. In order to solve the above problems, it is necessary to match the virtual coordinate system and the physical coordinate system. An accurate spatial transformation relationship is established between the two coordinate systems. The current real-time tracking methods can be mainly divided into two categories: one is synchronous, which can display the absolute motion of the surgical instruments and the patient lesions in the virtual space, and the other is to achieve tracking based on registration in the patient's lesion coordinate system [18].

**Fig. 3:**
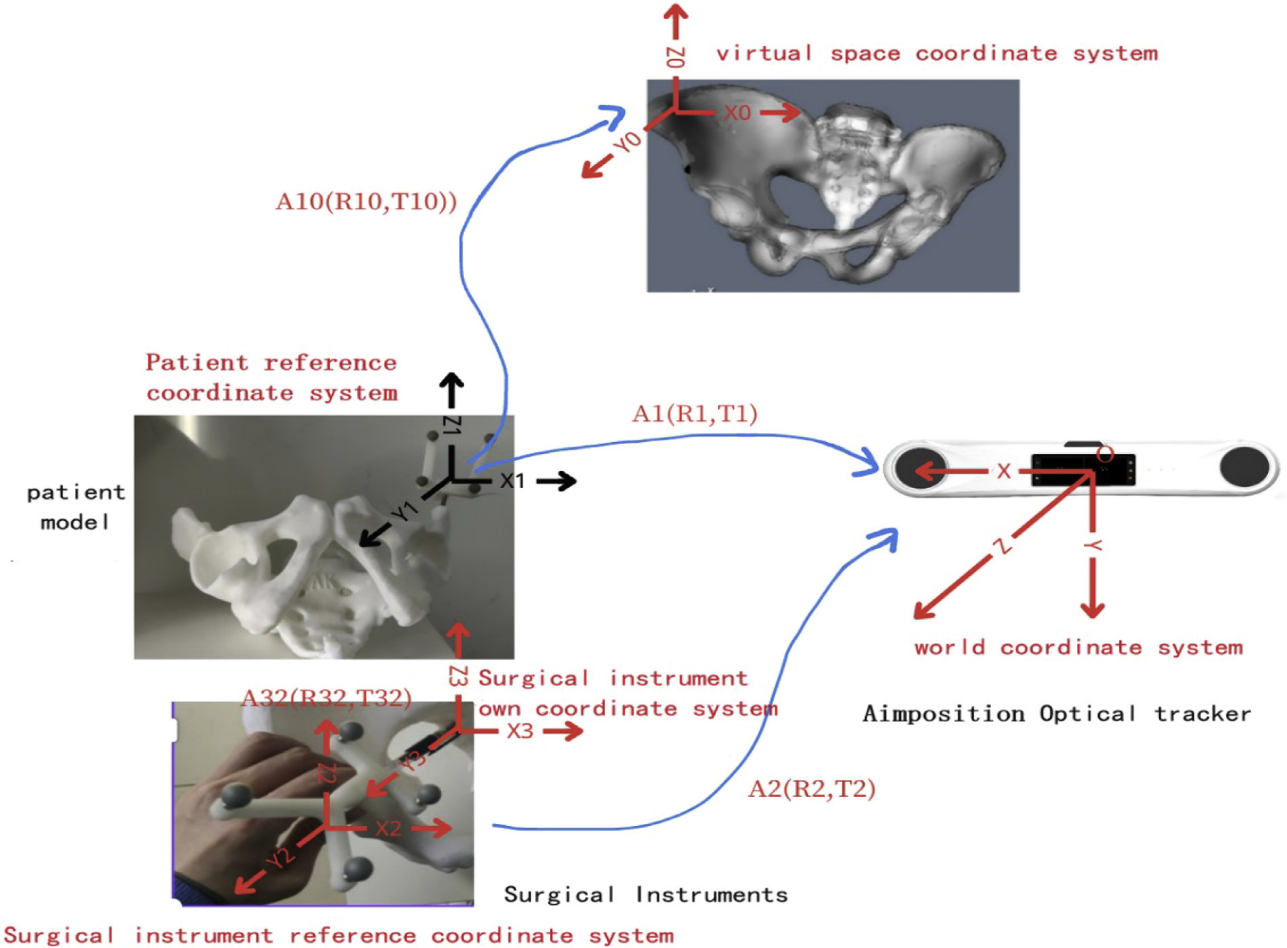
**Establishment of the coordinate systems for surgical navigation**. It is necessary to match the virtual coordinate system (i.e., the coordinate system formed on the computer screen after processing based on the pelvis model) and the physical coordinate system (i.e., world coordinate system, optical positioning system coordinate system, and the surgical instrument's own coordinate system).

This study used registration based on the patient's lesion coordinate system [19]. The basic registration process based on the patient's lesion coordinate system is to keep the lesion model stationary, track the relative motion of the surgical instrument, and follow the lesion model. This process reflects only the motion of the surgical instrument relative to the lesion, and its positional relationship with the computer screen accurately and consistently. Since the lesion model remains unchanged, the registration operation only needs to be run once. The aim of this method is to calculate the spatial transformation matrix related to the surgical instrument. The spatial coordinate system established by the surgical navigation system is shown in Fig. 3. The AimPosition optical positioning and tracking system is the OXYZ coordinate system. Since the optical positioning tracker expresses the OXYZ coordinates of the actual object in the form of world coordinates, it can also be expressed as the world coordinate system. The virtual space coordinate system in the computer screen is $O_{0}X_{0}Y_{0}Z_{0}$. Since the three-dimensional pelvis model itself also has a corresponding coordinate system, it can be set to the same $O_{0}X_{0}Y_{0}Z_{0}$ coordinate system to facilitate the subsequent operation process [20]. The positioning of the pelvic model by the optical positioning and tracking instrument was conducted using the calibration ball; therefore, the patient's $O_{1}X_{1}Y_{1}Z_{1}$ reference coordinate system was established based on the calibration ball on the pelvic model. In the same way, from the discussion of surgical instrument registration, it could be concluded that the optical positioning and tracking instrument binds the surgical instrument's $O_{2}X_{2}Y_{2}Z_{2}$reference coordinate system of the calibrated ball to the $O_{3}X_{3}Y_{3}Z_{3}$ coordinate system, with the tip of the surgical instrument as the origin.

The AimPosition optical positioning tracker can calculate the transformation matrices A1(R1,T1)andA2(R2,T2) between the calibration tool and its own coordinate system in real time when detecting the calibration ball. Thus, it can directly load $O_{1}X_{1}Y_{1}Z_{1}\;and\;O_{2}X_{2}Y_{2}Z_{2}$ into the relative OXYZ spatial transformation matrix (where R1andR2are the rotation matrices, and T1andT2 are the translation matrices).

For any point $P={\left(x,y,z\right)}^{T}$ in the world coordinate system, the points need to undergo coordinate transformation in four spaces: the tip of the surgical instrument, the fiducial of the surgical instrument, the pelvic model, and the virtual space. The transformation relationship is as shown in Eq. 5:

(5) $$P_{0}=\left[\begin{array}{cc} R_{10} & T_{10}\\ 0 & 1 \end{array}\right]{\left[\begin{array}{cc} R_{1} & T_{1}\\ 0 & 1 \end{array}\right]}^{-1}\left[\begin{array}{cc} R_{2} & T_{2}\\ 0 & 1 \end{array}\right]\left[\begin{array}{cc} R_{32} & T_{32}\\ 0 & 1 \end{array}\right]P_{3}$$

Among them, $P_{0}={\left(x_{0},y_{0},z_{0}\right)}^{T},\;P_{1}={\left(x_{1},y_{1},z_{1}\right)}^{T},\;P_{2}={\left(x_{2},y_{2},z_{2}\right)}^{T},\;$and $P_{3}={\left(x_{3},y_{3},z_{3}\right)}^{T}$, respectively, represent the coordinates of the P points in the virtual space, the pelvic model, the surgical instrument, and the surgical instrument's tip coordinate system [18]. Eq. 5 describes the parameters required to track the surgical instruments to correctly display the coordinates of the corresponding points in the virtual space. Obviously, the key parameters are the four spatial transformation matrices: matrix $A_{10}\left(R_{10},\;T_{10}\right)$, $A_{1}\left(R_{1},\;T_{1}\right)$, $A_{2}\left(R_{2},\;T_{2}\right),\;and\;A_{32}\left(R_{32},\;T_{32}\right)$. Among them,$\;A_{1}\left(R_{1},\;T_{1}\right),\;A_{2}\left(R_{2},\;T_{2}\right),\;and\;A_{32}\left(R_{32},\;T_{32}\right)$ can be obtained from our previous work. At present, surgical navigation requires the one-to-one mapping of points in the real pelvic model to match the points in the virtual space, which can meet the transformation needs of the pelvic model. The key lies in the $O_{0}X_{0}Y_{0}Z_{0}$ and $O_{1}X_{1}Y_{1}Z_{1}$ systems’ corresponding relationship with the $A_{10}\left(R_{10},\;T_{10}\right)\;spatial\;coordinate\;system$. In order to maintain the pelvic motion consistent in the two coordinate systems, registration work is required. To complete the registration, it is necessary to use the singular value decomposition SVD method to solve the registration matrix. Assume that the $O_{1}X_{1}Y_{1}Z_{1}$ coordinate system and the corresponding $O_{0}X_{0}Y_{0}Z_{0}$ point coordinate set on the coordinate system are, respectively, $P=\{P_{i},\;i=0,1,2,\ldots ,n\}$and $Z=\{Z_{i},\;i=0,1,2,\ldots ,n\}$, then:

(6) $$P=R_{10}*Z+T_{10}$$

In order to calculate $A_{10}\left(R_{10},\;T_{10}\right)$, it is necessary to know the $O_{1}X_{1}Y_{1}Z_{1}$ coordinates of n corresponding points between the patient's $O_{0}X_{0}Y_{0}Z_{0}$ lesion reference coordinate system and the virtual space coordinate system and perform fitting. From the corner point detection results presented in Section 3.2, the A, B, C, and D coordinates of the sphere center in the virtual space coordinate system in the pelvic model calibration ball were obtained, and their corresponding coordinates in the patient's lesion reference coordinate system can be obtained in real time using AimPosition [21]. Using Eq. 6, four equation systems can be concatenated, and the coordinate dimension was 3. Thus, the number of equation systems is greater than the coordinate dimension. This equation system is an overdetermined equation system and can be calculated using the SVD method [15].

Assume that the three-dimensional coordinates of the corresponding landmark points in the two coordinate systems are $P_{i}={\left(P_{xi},\;P_{yi},\;P_{zi}\right)}^{T}$and $Z_{i}={\left(Z_{xi},\;Z_{yi},\;Z_{zi}\right)}^{T}$; then, the registration objective function can be optimized using Eq. 7:

(7) $$F=\sum {\left|\left|P_{i}-\left(R_{10}Z_{i}+T_{10}\right)\right|\right|}^{2}\to min$$

Assuming that the centers of gravity of the point sets P and Z are $C_{P}=\;\frac{1}{n}\sum_{i=0}^{n-1}P_{i}$ and $C_{Z}=\frac{1}{n}\sum_{i=0}^{n-1}Z_{i}$, respectively, the covariance matrix C of the two point sets was calculated using the following formula:

(8) $$C=\sum_{i=0}^{n-1}\left(Z_{i}-P_{i}\right){\left(Z_{i}-P_{i}\right)}^{T}=\sum_{i=0}^{n-1}Z_{i}P_{i}-C_{Z}C_{P}$$

Let us denote the trace of C as the sum of the diagonals of the covariance matrix. To define the symmetric matrix U:

(9) $$U=\left[\begin{array}{cccc} Trace\left(C\right) & C_{12}-C_{21} & C_{20}-C_{02} & C_{01}-C_{10}\\ C_{12}-C_{21} & 2C_{00}-Trace\left(C\right) & C_{01}+C_{10} & C_{20}+C_{02}\\ C_{20}-C_{02} & C_{01}+C_{10} & 2C_{11}-Trace\left(C\right) & C_{12}+C_{21}\\ C_{01}-C_{10} & C_{20}-C_{02} & C_{12}+C_{21} & 2C-Trace\left(C\right) \end{array}\right]$$

The calculation of the eigenvalues and eigenvectors of the matrix U (the Jacobi method can be used) can lead to the eigenvector corresponding to the maximum eigenvalue [22,23]. This eigenvector is the rotation quaternion that satisfies the minimum value of F in Eq. 7, and then the corresponding rotation matrix R30 can be obtained. The translation vector can be obtained from the center of gravity of the two point sets by the following formula:

(10) $$T_{10}=C_{P}-R_{10}C_{Z}$$

The specific implementation process is shown in Fig. S2

### 2.6 Design and implementation of the minimally invasive surgical navigation system of pelvic channel screws

The function of this system is to track changes in the relative positions of the surgical instruments and pelvic models in real time based on the visual information provided by the optical positioning tracker. Fig. S3 is a case diagram that shows the interaction requirements between the users and the system or between the components within the system.

When users apply the system, they need to perform the following four steps. The first step is to set the virtual space coordinates for the pelvic model calibration ball in the system. The user needs to obtain the coordinates through the 3D corner detection system and input the data into the system to obtain the coordinate results [9]. The second step is to import the pelvic model. The pelvic model of the system at this stage is an STL file, which is obtained by the three-dimensional reconstruction of the patientʼs pelvis. The third step is to prepare the surgical instruments and complete the calibration of the surgical instruments. Under normal circumstances, the strict structural relationship between the surgical instruments and the calibration ball cannot be changed; therefore, the surgical instrument usually only needs to be calibrated once during the process of the entire system. The fourth step is to start the optical positioning instrument for tracking, as shown in Fig. 3. The system extracts the calibration's spherical coordinates in the positioning instrument and solves the mapping matrix, using this mapping matrix to correctly reflect the relative motion of the pelvic model and surgical instruments on the screen.

**Fig. 4:**
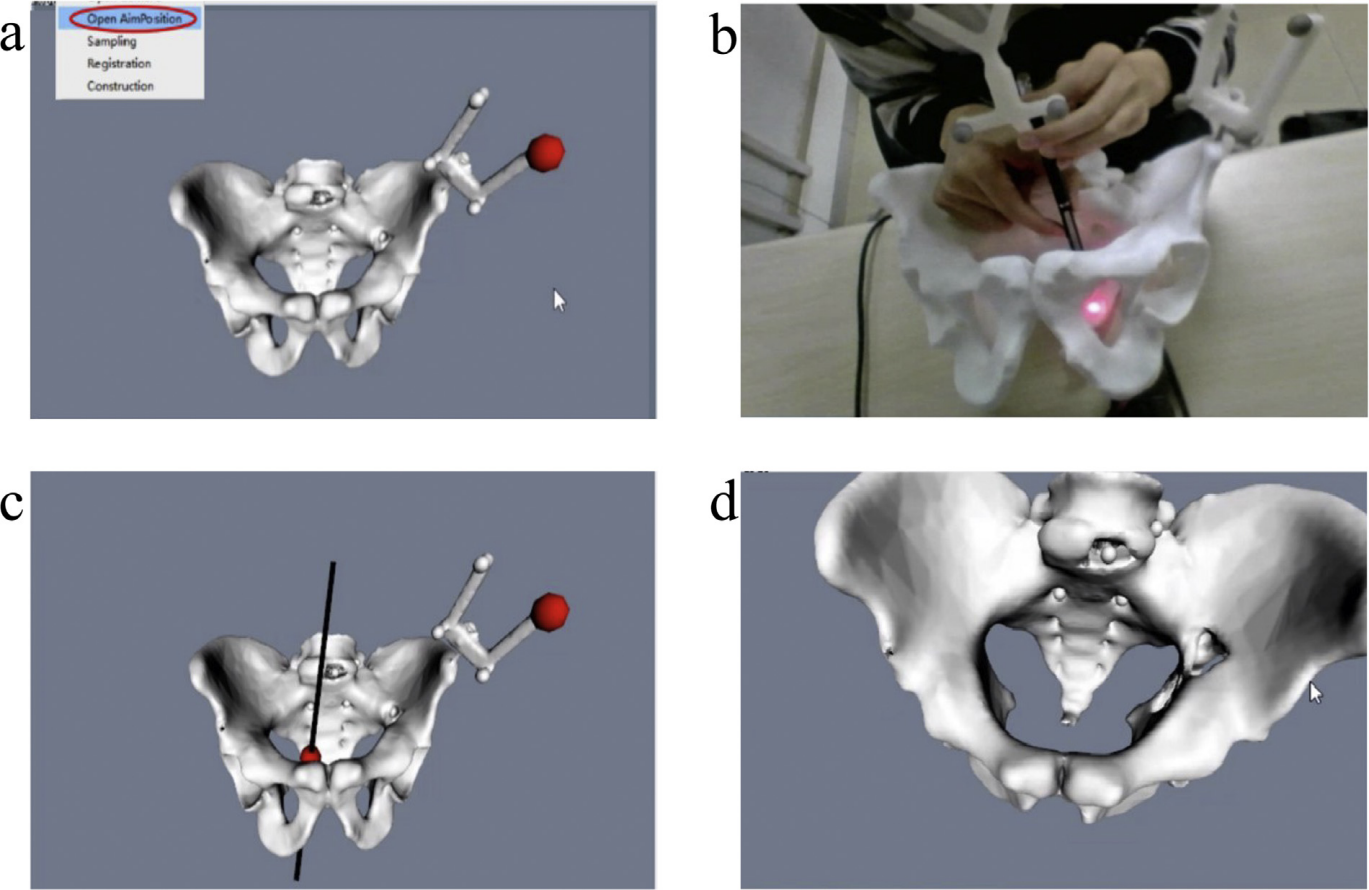
**Software interface of the surgical navigation system**. (a) The pelvis model in AimPosition. (b) The real position of pelvis model. (c) The direction of the tip. (d) The view perspective of the tip direction.

As shown in Fig. 4, firstly, the AimPosition optical positioner starts, and then the user needs to click the “Open AimPosition” UI button under the action bar to open the interface between the program and the optical positioner. The pelvis model and the positioning ball of the surgical instrument are placed within the field of view of the optical positioner. At this point, the surgical navigation function is successfully activated. After starting, the system automatically tracks the position of the surgical instrument.

### 2.7 Experimental environment and statistical analysis

The computer used for the surgical navigation was a 64-bit Windows 10 workstation. The relevant experimental environment hardware included the following: the processor was Intel (R) Core (TM) i7–3970 3.50Ghz, the graphics card was NVIDIA GeForce GT720, and the memory was 16GB. An AimPosition infrared optical positioning device was used to track the movement of the surgical instruments (replaced by a laser pointer) and four markers were attached to the needle handle. The laser pointer was 16 cm long and the diameter of the marking point ball was 1.2 cm.

In terms of code implementation, this research selects the C++ 14 standard as the development language for the minimally invasive surgical navigation system to implement the pelvic channel screws. In terms of the implementation of the main logic, the third libraries it relied on included the matrix calculation library Eigen3 and the point cloud processing library PCL1.8.1; in terms of graphics rendering and GUI interaction, the system relied on the visualization tool package VTK8.0.0 and the graphical user interface framework Qt5.9.4. Statistical data analysis was completed using SPSS18.0.

## 3 Results

### 3.1 Surgical instrument registration results

In the minimally invasive surgical navigation system to implement pelvic channel screw, the calibration accuracy of the surgical instrument tip is particularly important for the accuracy of the surgical effect. Therefore, experiments needed to be designed to verify the accuracy of the above method for the calibration of the surgical instrument's tip. The experiment consisted of two parts; one was the RMSD analysis of the X offset of the calibration results, and the other part was the analysis of the distance error between the position of the surgical instrument's tip and each marker ball.

It can be observed from Formulas 2.3 and 2.4 that the offset from the tip of the surgical instrument to the $O_{2}X_{2}Y_{2}Z_{2}$ coordinate system of the calibration ball was maintained $X_{w}$ unchanged and then rotated to obtain the rotation and translation matrices at different positions; thus, the results obtained at each position were averaged. Therefore, in this experiment, the world coordinates of the surgical instrument tip $X_{w}$ were changed. The initial surgical instrument's tip was fixed at 10 different positions; the tip was calibrated at these positions, and the offset RMSD at different initial positions was calculated. The calculation results are as shown in Table 1.

**Table 1:** **RMSD error(mm) results of the calibration of the surgical instrument's tip**.

1	2	3	4	5	6	7	8	9	10
0.209	**0.126**	0.212	0.158	0.189	0.165	0.182	**0.215**	0.203	0.193

It can be observed from Table 1 that the calculated offset was accurate and there was no considerable fluctuation error. The largest RMSD error was 0.215 mm, the smallest RMSD error was 0.126 mm, and the average RMSD error was 0.185 mm, which was less than 0.3 mm [8]. After conducting a *t*-test, we obtained a *p*-value < 0.01 (*N* = 10), while the general surgical error was greater than 0.3 mm. Therefore, this method was vastly superior to the accuracy requirements of general surgical navigation systems and has extremely high potential and application value.

We used the calibration results to which the RMSD error median belong for the subsequent experiment. After calibration, the AimPosition could not only load the world coordinates of the four calibration balls, but also calculate and load the world coordinates of the tip of the surgical instrument. We assumed that the $O_{1}$ distance from the tip of the surgical instrument to the calibration of the A,B,C,and D balls are $r_{A},\;r_{B},\;r_{C}$, and $r_{D}$ respectively; the distances from the tip of the surgical instrument are shown in Figure S4. The accuracy of calculation of the tip position was obtained by using the distance from the tip position of the surgical instrument to the centers of the four calibration balls.

As shown in Figure S4, the surgical instruments were placed in any five positions, and a ruler was used to calculate $r_{A},\;r_{B},\;r_{C}$, and $r_{D}$, with the average being the real physical distance. The calculation results are $r_{A}$ = 186.93 mm, $r_{B}$ = 184.87 mm, $r_{C}$ = 247.91 mm, and $r_{D}$ = 255.36 mm. Then, we placed the surgical instrument in any 10 positions. The world coordinates of the tip of the surgical instrument and A,B,C,and D were calculated using the distance from the tip of the surgical instrument to A,B,C,and D, according to the distance formula, and were recorded as $r_{Ai}$, $r_{Bi}$,$\;\;r_{Ci}$, and$\;r_{Di}\left(i=1,2,3,\ldots ,10\right)$, respectively. The calculation results are shown in Table 2. The smallest difference corresponded to $|r_{Bi}-r_{B}|$, which was 0.02 mm from $r_{B}\;(Figure\;$S4), and the largest difference corresponded to $|r_{Ci}-r_{C}|$, which was 2.13 mm from $r_{C}$ (FigureS4). The average errors of $|r_{Ai}-r_{A}|$, $|r_{Bi}-r_{B}|$, $|r_{Ci}-r_{C}|$, and$\;|r_{Di}-r_{D}|$ were 1.003 mm, 1.111 mm, 1.140 mm, and 1.075 mm, respectively. After conducting an analysis of variance F-test, we obtained a *p*-value >0.05, which meant that there is no significant difference between them.

**Table 2:** **Distance values(mm) between the instrument's tip and the calibration point according to Fig. S4**.

	$|r_{Ai}-r_{A}|$	$|r_{Bi}-r_{B}|$	$|r_{Ci}-r_{C}|$	$|r_{Di}-r_{D}|$
1	0.32	0.66	1.46	1.85
2	0.76	0.26	1.14	1.04
3	1.92	1.59	1.17	0.54
4	1.69	1.91	1.10	0.61
5	1.84	0.93	1.55	1.96
6	1.07	0.48	0.15	0.99
7	0.27	1.96	0.50	1.35
8	0.40	1.48	0.57	0.45
9	0.32	1.82	1.63	0.13
10	1.44	**0.02**	**2.13**	1.83

### 3.2 3D Harris algorithm selection results for marked balls

**Fig. 5:**
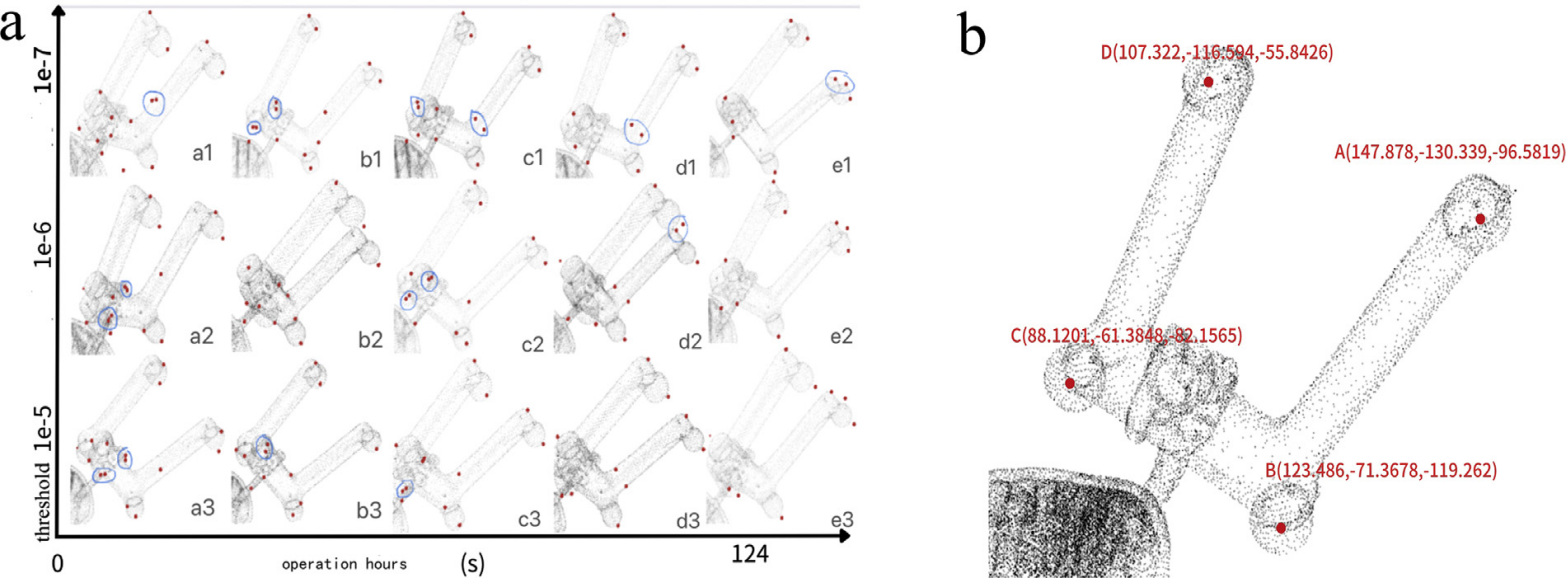
**Surgical instrument registration and 3D Harris algorithm selection results**. (a) Corner point detection effect diagram under different parameter settings. (b) Calibration ball coordinates obtained using corner point detection.

Firstly, a series of parameter values were used for testing, and the detection results were compared. Finally, the best suitable parameters for this model were compared. After the pelvis model STL file was converted into point cloud data, there were 439,033 points in total. The number of generated point clouds was large and the density was high. As the radius size of the neighborhood sphere cannot be too large, this study set the radius size value to 8, 9, 10, 11, and 12, and the non-maximal and suppression threshold was tested using 10^−7^, 10^−6^, and 10^−5^. The corner detection effect is shown in Table S1 and Fig. 5a. It can be observed that the smaller the radius of the detection ball and the lower the threshold setting, the more corner points are obtained and the detection reaction time reduced. The relationship between the two was *y* = 128.68219–0.36620x (*x* = number of corner points, *y* = detection time, *p*-value < 0.01, R2 = 0.65).

In Fig. 5a, the detected corner points are represented in red, and possible false corner points ae represented in blue circles. It can be observed from Fig. 5a and Table S1 that, when the sphere radius was set to 9 and the threshold was set to 10^−6^, the effect was suitable and the detection time was moderate. The center coordinates of the four calibration balls A,B,C,andD can be observed in the visual interface. The virtual coordinates obtained by loading were A (147.878, −130.339, −96.5819), B (123.486, −71.3678, −119.262), C (88.1201, −61.3848, −82.1565), and D (107.322, −116.594, −55.8426), and the specific relationship is shown in Fig. 5b.

The ultimate effect of surgical navigation displayed on the computer screen is to track the position of surgical instruments in real time. Their motion relationship was accurately displayed in the pelvis model, and the surgical path was predicted based on the position of the surgical instruments, thereby guiding the surgery and improving the accuracy. Therefore, the most critical indicator to evaluate the performance of surgical navigation system is its own accuracy. Errors in the surgical navigation system included systematic and accidental errors. This research did not consider accidental errors, only systematic errors. Registration errors are the main cause of systematic errors. Therefore, this project designed an experiment to detect the distance error based on the SVD registration method using the manual setting comparison method and verified the effectiveness of the solution.

### 3.3 Results of the path prediction in pelvic model experiment

To conduct model experiments to verify the accuracy of the predicted route of the surgical instruments, four additional calibration balls were placed on the pelvic model. Since the relative spatial relationship between the surgical instrument and the pelvic model remains unchanged after registration, if the tip of the surgical instrument is aligned with the calibration ball, the line segment relationship between the two should also remain unchanged. Based on this principle, the predicted surgical path was set as the intersection point of the extension line of the tip of the surgical instrument and the pelvic model. The surgical instruments were placed at different positions of the pelvic model. The point of the calibration ball and the predicted route of the surgical instrument were calculated in the virtual space. The distance error was set as the distance between the marked points on the pelvis. The above experimental procedure was designed by the manual setting comparison method, which was used to calculate the average and standard deviation of the positioning error of the experimental surgical instrument (laser pen) at different positions.

**Fig. 6:**
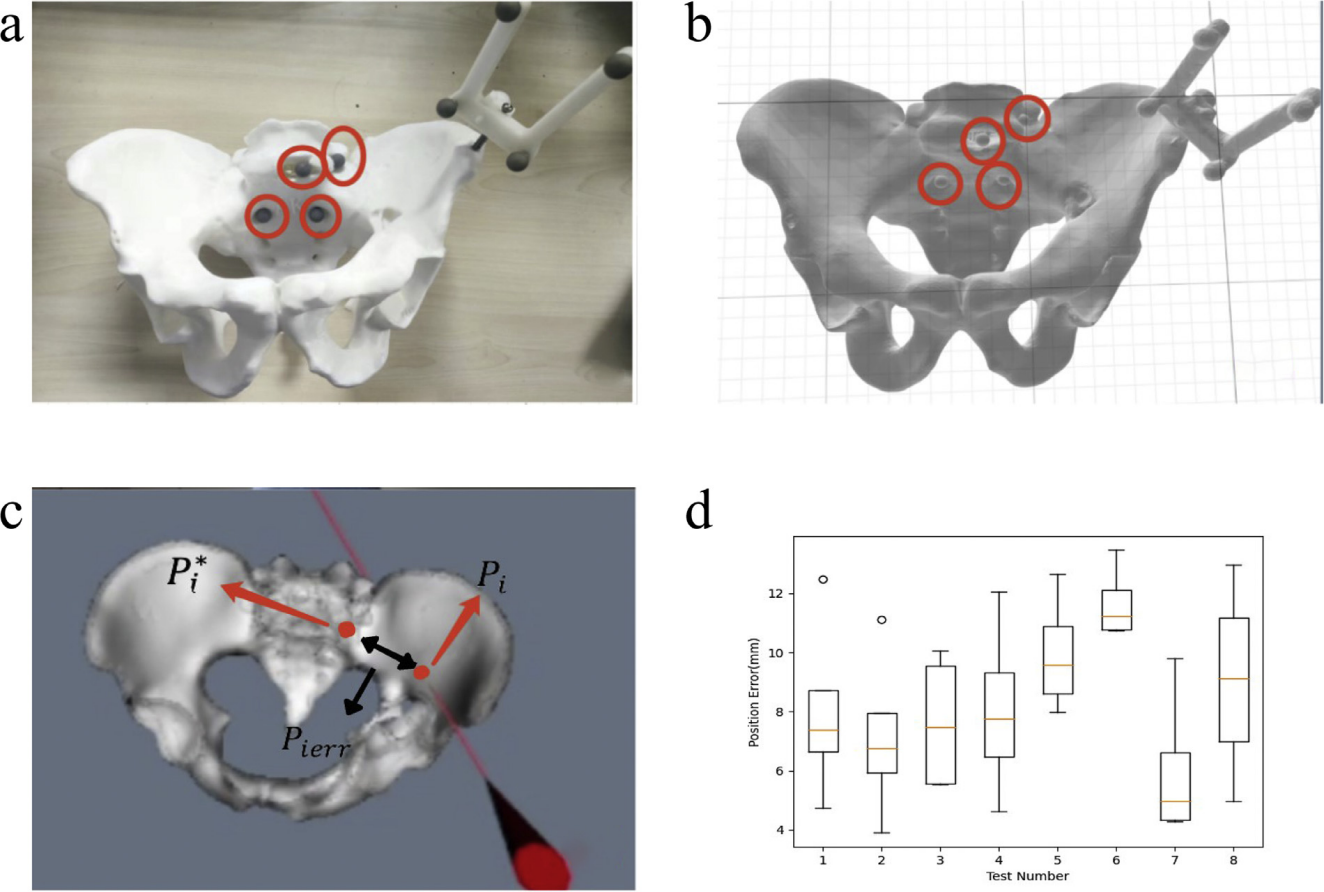
**Schematic diagram of the registration error experiment**. (a) Infrared reflective balls placed on the surgical tool and pelvic model. (b) Tips of the surgical instruments on the pelvis. (c) Laser pointer and the pelvic model. (d) Statistical chart of the registration error using a two-tailed F-test, *p*-value > 0.05.

In order to ensure that the navigation accuracy during the actual surgery was consistent with the navigation accuracy that was verified by this experiment, the laser pen was placed to simulate the process of the doctor adjusting the surgical instruments during the surgery, as shown in Fig. 6. To increase the accuracy and validity of the test results, the specific steps are described below and shown in Fig. 6:

(a) Install the positioning and tracking fiducial with the infrared reflective ball on the surgical tool and pelvic model, so that it can be positioned and tracked using the AimPosition optical camera in real time.

(b) Calibrate the tips of the surgical instruments, as described in Section 2.

(c) Select four reference points according to the pelvis model. The STL file of the model with the installed reference points is regenerated and displays the corresponding reference points in the three-dimensional model in the virtual image. This is shown in Figs. 6a, b.

(d) Use the laser pointer to select the datum points on the pelvic model in sequence and align the tip with the datum point, as shown in Fig. 6c. The $\;P_{i}\;\left(P_{ix},\;P_{iy},P_{iz}\right)$ intersection position of the laser pointer tip ray with the pelvic model in the virtual image is recorded, and then its intersection with the modeling point is calculated. The $P_{ierr}$ distance error between the theoretical positions of the reference points $P_{i}^{*}\;\left(P_{ix}^{*},P_{iy}^{*},P_{iz}^{*}\right)$ is shown in Fig. 6c, and the calculation formula is as follows.

(11) $$\begin{array}{ccc} P_{ierr} & = & \sqrt{{\left(P_{ix}-P_{ix}^{*}\right)}^{2}+{\left(P_{iy}-P_{iy}^{*}\right)}^{2}+{\left(P_{iz}-P_{iz}^{*}\right)}^{2}\;},\\ & & \left(i=1,\;2,\;3,\;\ldots ,\;20\right) \end{array}$$

Repeat the four steps mentioned above to calculate the distance error of the pelvic surgery navigation system, as designed in this research. The experiment was conducted according to the above steps, and the results are shown in Table 3 and Fig. 6d.

**Table 3:** **Results of the registration's effect distance error (mm)**.

Experiment ID	Mark point ID				Average error
	1	2	3	4	
1	7.46	4.74	7.28	12.48	7.99
2	6.92	11.10	6.61	3.90	7.13
3	10.07	5.54	5.58	9.36	7.63
4	8.40	7.08	4.61	12.06	8.03
5	7.97	10.31	12.65	8.82	9.93
6	11.67	10.74	13.46	10.77	**11.66**
7	**4.37**	4.28	9.80	5.57	**6.01**
8	4.96	**12.97**	10.56	7.65	9.04

As shown in Fig. 6d, the average error of the registration results for the eight groups of experiments was 8.43 ± 1.34 mm. After conducting an analysis of variance with a two-tailed F-test, we obtained a *p*-value > 0.05, which indicates that the method has good repeatability, accuracy, and practical application value.

## 4 Discussion

According to the data in Table 2, it can be clearly observed that the calibrated calculated position of the surgical instrument's tip is consistent with the actual physical distance. The analysis of the calibration error results shows that the maximum error was only 2.13 mm and the minimum error was 0.02 mm. The average error was 1.08 mm. This shows that the average distance error between the tip of the surgical instrument and the calibration ball during the operation is approximately 1.08 mm. In a common surgery procedure, this error can be tolerated [24]. This method, therefore, presents extremely excellent results. This error could be attributed to the fact that the default tip position during the experiment is a point in three-dimensional space. However, in fact, the tip of the surgical instrument is closer to a sphere, and its own diameter is not negligible. In addition, the surgical instrument will rotate around the tip during the measurement process, which leads to the inability of maintaining the tip position completely stationary. Even a slight movement may cause changes in the values of Eq. 2, which leads to larger errors.

Based on the above analysis, two feasible solutions to reduce errors are proposed. First, if the surgical tip is treated as an approximate sphere, and therefore its diameter is reduced, the calculation accuracy is improved. Therefore, more accurate calibration algorithm results can be obtained by minimizing the diameter of the surgical tip. Secondly, the stability in Eq. 2 needs to be ensured. The tip position should be maintained at a fixed position as much as possible during the rotation of the surgical instrument. This can be achieved through the physical method, such as fixing the tip to a rough plate to increase the stability of the tip position. Furthermore, in order to obtain more accurate fitting tip position data, we recommended collecting as many data as possible during the rotation. Although reducing the tip of the ball to a certain extent objectively increases the cost and complexity of the operation, it greatly reduces the scanning time and increases the number and sensitivity of corner detection, which still has considerable practical significance.

In addition, according to the experimental results of the 3D Harris parameters in Table S1 and Fig. 6, it can be observed that the non-maximum suppression threshold has a significant impact on the corner detection results. A threshold that is too large may imply few corner points, while a threshold that is too small that may increase the number of false corner points. For the radius size of the spherical space region, choosing a smaller sphere radius can speed up the calculation of the points required for the normal vector, but it may also result in an excessive number of corner points, including many pseudo-corner points. Choosing a larger sphere radius will reduce the number of corner points, but it will also slow down the calculation and may cause some corner points to be missed. We suggest that future research addresses how to achieve a better balance, both to ensure that there are enough effective detection corners and to remove too many useless and pseudo-corners.

Therefore, in this study, the manual setting comparison method was used to assess 3D corners as a suitable detection parameter for this shape, which can effectively solve the above problems. However, it should be pointed out that the manually set threshold is not universal, and the same threshold may have different effects measured in different point cloud data. Considering the versatility of the algorithm, it is not practical to set different thresholds for each set of point cloud data. Therefore, it is still necessary to gradually explore and propose more suitable methods in future work.

In Table 3, the accuracy of predicting the surgical route intersection points is shown. The errors of each point within the groups are relatively consistent, and there are some differences in the errors between groups. The minimum value was 6.01 mm. The maximum value was 11.06 mm, and the average error was 8.43 ± 1.34 mm. And Martin et al. [25] conducted experimental results with similar errors. Since the current tip position, screw insertion position, and perspective pelvic structural bones are visualized in the navigation system, doctors can observe the length of the screw insertion and the angle with the sagittal plane and transverse plane in real time, effectively controlling and ensuring safety within a stable range. The above solution is sufficient to ensure that the virtual reality surgical method developed by this research institute is far superior to the traditional surgical solution and allows doctors to perform surgeries more effectively and safely. Thus, it should be widely used in clinical practice.

## 5 Conclusions

Along with the registration principle based on the patient coordinate system, this article applied the manual setting comparison method to select suitable 3D corner detection parameters for the augmented reality surgery technology. This method can effectively reduce the experimental error in augmented reality surgery. Compared with the traditional methods, it also can achieve more precise and safer minimally invasive pelvic surgery results, which is of great value and deserves further attention in the clinic.

## Declaration of competing interest

The authors declare that they have no conflicts of interest in this work.

## Appendix Supplementary materials

Image, application 1
