# Supplementary material for: Augmented reality navigation for precise implantation of LC2 pelvic tunnel screws in minimally invasive surgery
Source: Fundam Res. 2024 May 28;6(1):338–46. doi: 10.1016/j.fmre.2024.05.010 (PMC12869777; doi:10.1016/j.fmre.2024.05.010)
Supplement: Supplementary file 1 [file mmc1.docx]

**Table S1. Corner detection effects under different parameter settings**

| Radius size | Serial number | Threshold | Number of corner points | Detection time(s) |
| --- | --- | --- | --- | --- |
| 8 | a_1_ | 10^-7^ | 165 | 76.5 |
|  | a_2_ | 10^-6^ | 160 | 76.9 |
|  | a_3_ | 10^-5^ | 157 | 76.8 |
| 9 | b_1_ | 10^-7^ | 97 | 82.1 |
|  | b_2_ | 10^-6^ | 93 | 82.8 |
|  | b_3_ | 10^-5^ | 89 | 81.9 |
| 10 | c_1_ | 10^-7^ | 92 | 90.5 |
|  | c_2_ | 10^-6^ | 88 | 90.9 |
|  | c_3_ | 10^-5^ | 85 | 90.6 |
| 11 | d_1_ | 10^-7^ | 64 | 102.9 |
|  | d_2_ | 10^-6^ | 61 | 102.5 |
|  | d_3_ | 10^-5^ | 57 | 102.8 |
| 12 | e_1_ | 10^-7^ | 54 | 125.3 |
|  | e_2_ | 10^-6^ | 50 | 124.8 |
|  | e^3^ | 10^-5^ | 48 | 124.9 |


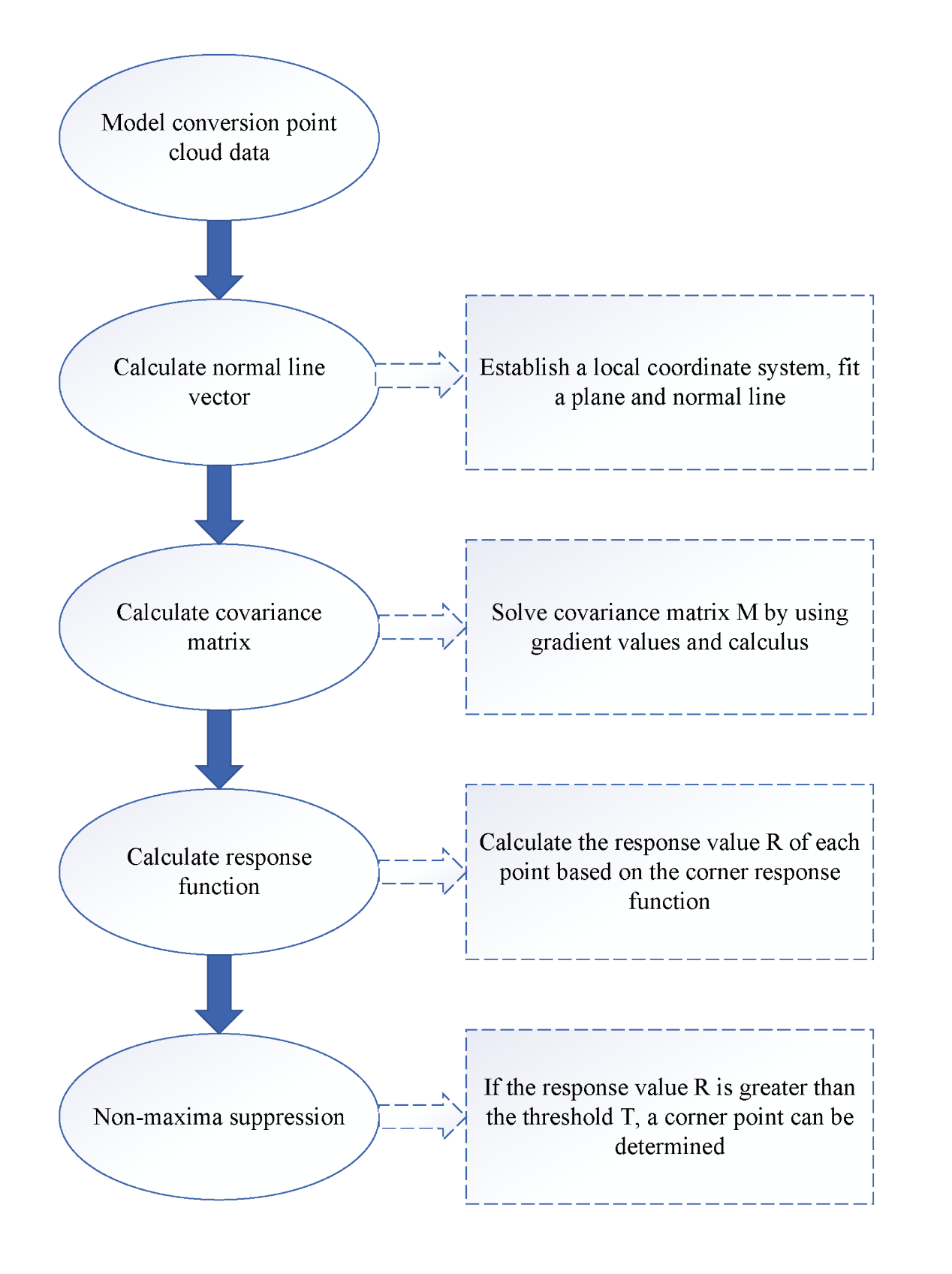


**Fig. S1**. 3D Harris algorithm flow chart. The 3D Harris algorithm recognition method is used to register the model in the virtual space in line with the real space.


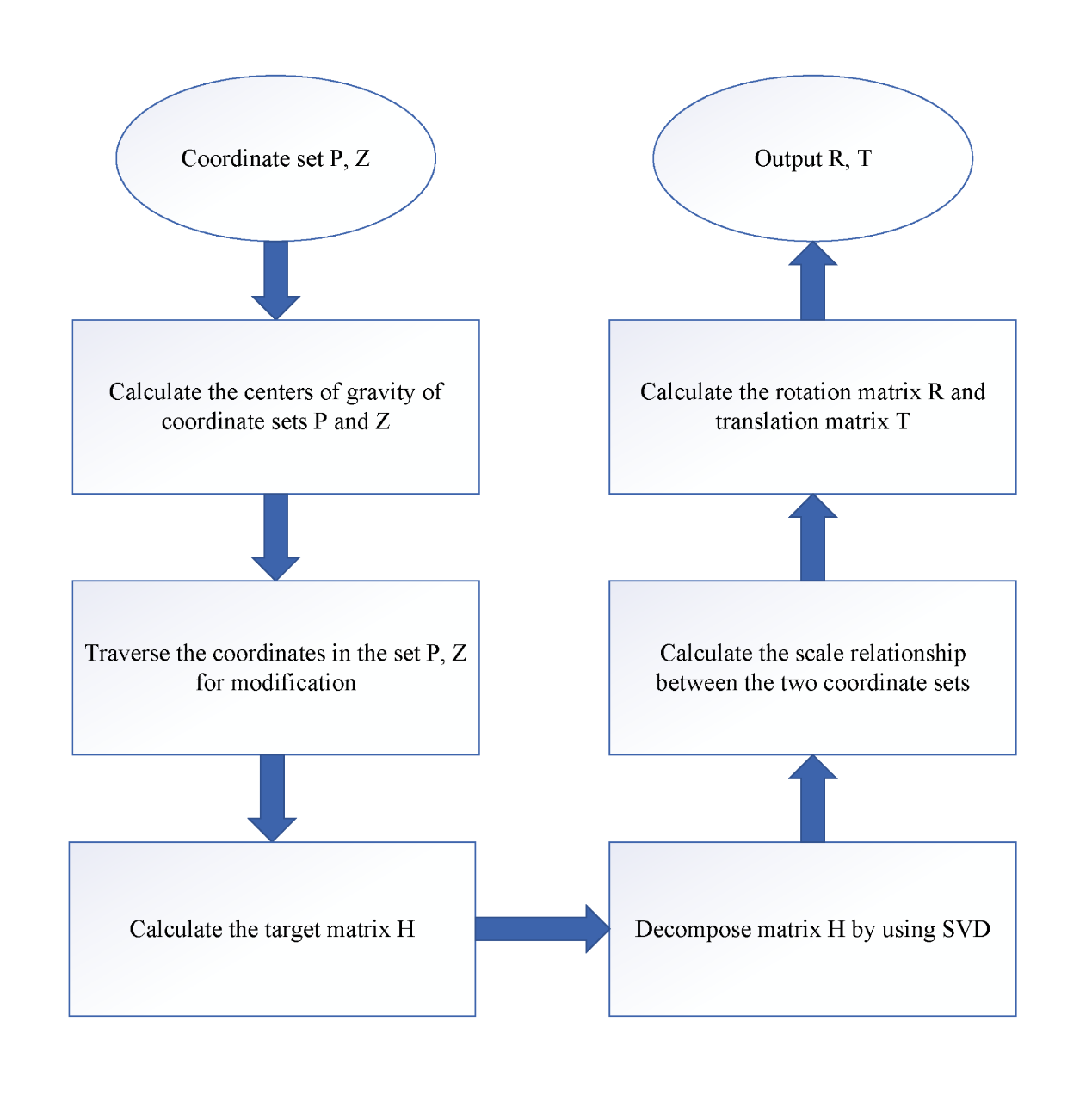


**Fig. S2**. The implementation process of the AimPosition coordinate system.


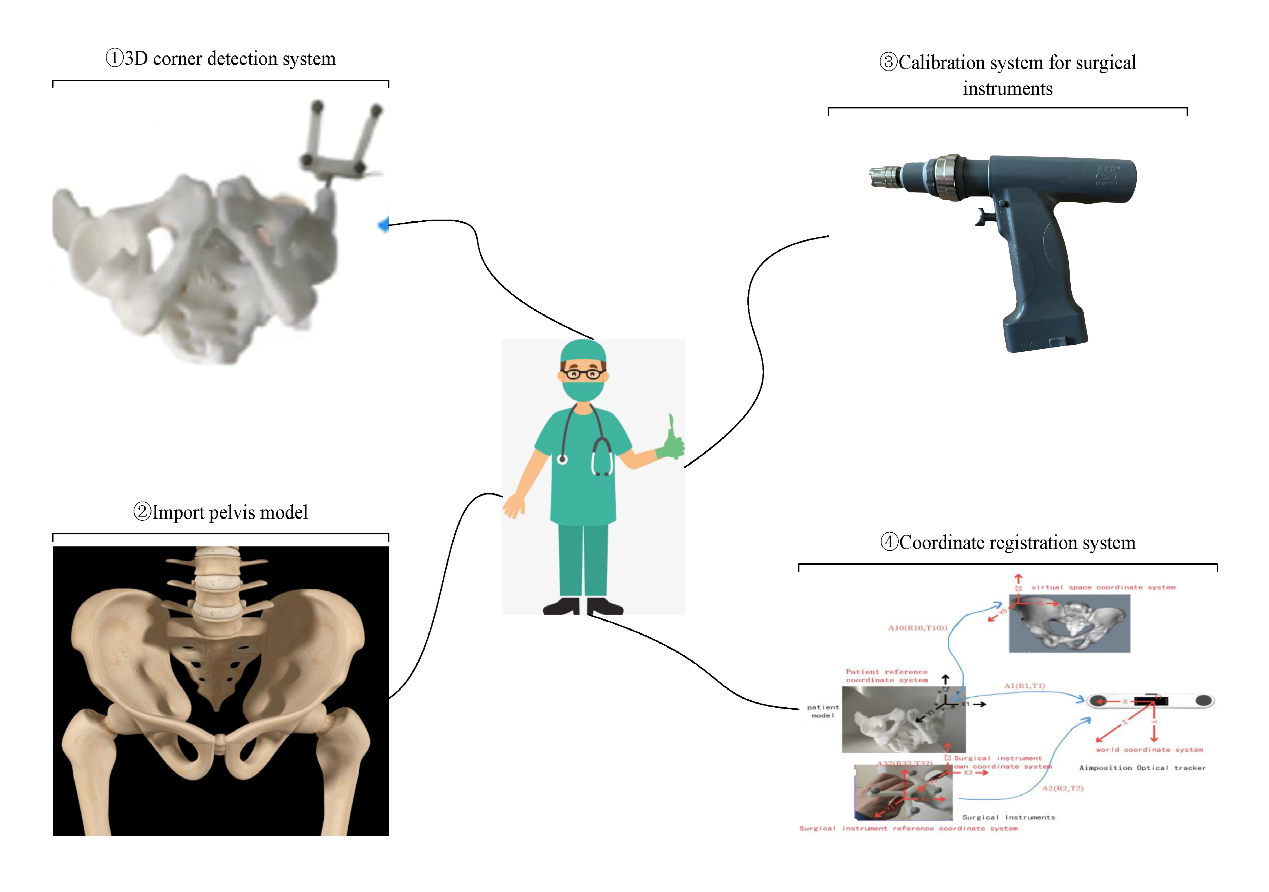


**Fig. S3**. Case diagram of the system. The application of this system requires the following four steps: The first step is to set the virtual space coordinates for the pelvic model calibration ball in the system. The second step is to import the pelvic model. The third step is to prepare the surgical instruments and complete the calibration of the surgical instruments. The fourth step is to start the optical positioning instrument for tracking, as shown in Figure 3.


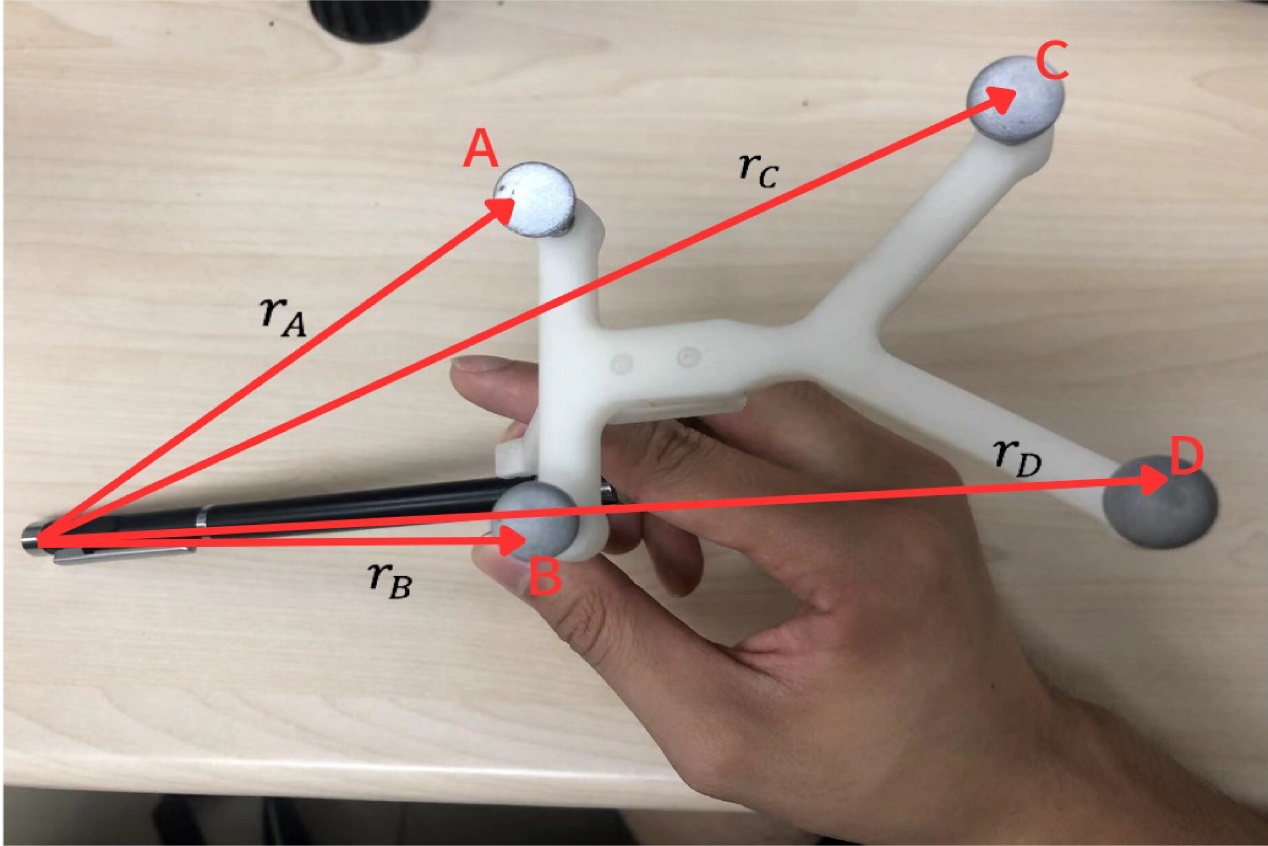


**Fig. S4.** Distance between the instrument’s tip and the calibration point.
